# Pollen transcriptomic analysis provided insights into understanding the molecular mechanisms underlying grafting-induced improvement in potato fertility

**DOI:** 10.3389/fpls.2024.1338106

**Published:** 2024-03-28

**Authors:** Xing Zhang, Lei Bai, Maoxing Li, Youhan Li, Ronghai Hu, Huachun Guo

**Affiliations:** ^1^ College of Agronomy and Biotechnology, Yunnan Agricultural University, Kunming, Yunnan, China; ^2^ Yunnan Engineering Research Center of Tuber and Root Crop Bio-breeding and Healthy Seed Propagation, Yunnan Agricultural University, Kunming, Yunnan, China; ^3^ Tuber and Root Crops Research Institute, Yunnan Agricultural University, Kunming, Yunnan, China; ^4^ Technical Department, Yunnan BengLong Potato Planting Co., Ltd, Kunming, Yunnan, China

**Keywords:** potato, grafting, fertility, pollen transcriptomics, carbohydrate metabolism

## Abstract

**Introduction:**

Heterologous grafting has been proven to be a valid approach to improving potato fertility, especially when grafting potatoes with other Solanaceae family plants. However, the mechanisms underlying grafting-induced improvement in potato fertility are still unknown.

**Methods:**

In this study, a poor-fertility potato cultivar “Qingshu No. 9” (Q9) was grafted with a tomato cultivar “Zhongyan988” (ZY988) to study the effects of heterologous grafting in the former. The tuber yield was controlled by different grafting and cultivation approaches, and the correlation between tuber yield and pollen vigor was studied. Comparative transcriptomic analysis of the potential mechanisms of pollen in potato scion fertility changes.

**Result:**

Grafting with the tomato rootstock effectively promoted the flower and fruit formation in the scion potato and improved its pollen viability by 15%–20%. In addition, a significant negative correlation was observed between the potato tuber yield and pollen viability, suggesting a potential impact on the metabolic regulatory network related to tuber formation. From the comparative transcriptomic analysis between the pollens from Q9 self-grafted plants and Q9-tomato grafting scion, 513 differentially expressed genes (DEGs) were identified. These DEGs were found to be related to gametophyte and pollen development, carbohydrate metabolism, and protein processing. Thus, these DEGs might be involved in improved fertility after reduced tuberization in plants subjected to heterologous grafting.

**Discussion:**

Potato/tomato heterologous grafting significantly improved the pollen viability of scion potatoes and was associated with the absence of potato tubers. Heterologous grafting promotes the transcription of genes related to protein processing, carbohydrate metabolism, and pollen development in pollen cells, resulting in the production of fertile pollen. Our results provided initial clues to understanding the improvement of potato fertility using the heterologous grafting method, which might be a useful tool in assisted potato breeding.

## 1 Introduction

Potato (*Solanum tuberosum* L.) is the third most widely consumed staple food crop worldwide. With the increase in consumption and the diversification of consumption methods, it is necessary for the breeders to continuously develop novel varieties to meet consumer needs (Dolničar, 2021). However, potato breeding is a slow process. This delay might be attributed to the generation of a low number of flowers and their poor blooming and pollen vitality in several cultivated varieties with excellent traits, such as “Russrt Burank” (McLean and Stevensen, 1952; Nassar et al., 2011) and “Qingshu No. 9” (Wang et al., 2022), making it difficult to use these varieties as parents for hybrid breeding.

Breeders try to address these issues by improving potato fertility. Artificial breeding techniques, such as extending the photoperiod to 18 h and manipulating the ambient temperature, can help achieve high fruit-setting rates. However, exposure to high temperatures leads to significant abscission of flower buds (Patterson, 1953). Moreover, potato tubers are strong assimilate sinks (Sweetlove et al., 1998), leading to potential competition for assimilation between floral organs and tubers (Werner, 1934; Almekinders and Struik, 1996). Some studies have shown that stolon pruning can stimulate the aboveground growth in plants and improve the flowering and pollen quality of certain varieties (Thijn, 1954; Jessup, 1958). However, some studies suggest that stolon removal enhances the growth of plants but does not impact flowering and pollen quality (Abdel-Wahab and Miller, 1963; Plantenga et al., 2019).

Furthermore, girdling cutting removes the stem cortex and use steel wire to tie the base and is used to limit the downward transport of carbohydrates, promoting the flowering and pollen quality in some varieties; however, it also affects plant growth (McLean and Stevensen, 1952; Thijn, 1954). In potato plants, several grafting techniques, such as grafting potato on tomato rootstocks, are used to enhance flowering and fruit-setting rates (Thijn, 1954; Jiang, 2016; Takeuchi et al., 2022). A previous study showed that *Physali alkekengi* grafting could effectively overcome the problem of hybrid parental infertility in potatoes (Qi, 2015). Another study reported that grafting “Qingshu No. 9” on *Datura stramonium* rootstocks can help obtain true potato seeds (Zhang et al., 2022). However, the mechanisms underlying enhanced flowering and fruiting in scion potatoes post-grafting are still unclear.

Another study on the “Russrt Burank” potato reported that ungrafted, self-grafting, and intermediate rootstock grafting did not affect flowering and fruit formation, indicating that grafting did not affect plant fertility. For instance, after the grafting of “Russrt Burank” potato with five wild potatoes, the flowering and fruit set of the “Russrt Burank” scion did not differ significantly between rootstocks with non-tuber-bearing habit and tuberizing rootstock (Weinheimer and Woodbury, 1966). Similarly, the *S. etuberosum* and *S. palustre* rootstocks with non-tuber-bearing habits did not promote the development of the CE3027 flower bud (Plantenga et al., 2019). Therefore, the presence of assimilation competition between floral organs and tuber remains controversial.

In this study, to elucidate the potential effects of grafting on the fertility of scion potatoes, we grafted “Qingshu No. 9” on two potato cultivars with excellent fertility and tomato rootstocks. In addition, we assessed the relationship between tuber and pollen activity. We used grafting techniques to control tuber yield and performed a transcriptome analysis of the potato pollen from a potato/tomato heterologous grafted scion to elucidate the mechanisms underlying graft-mediated enhancement in the fertility of scion potatoes. Our results demonstrated that heterologous grafting can promote the expression of genes related to protein processing and carbohydrate metabolism within the pollen cells, improving pollen fertility.

## 2 Materials and methods

### 2.1 Plant materials and growth conditions

Primarily, we used “Qingshu No. 9” (Q9, *S. tuberosum*), a potato variety that blooms normally but does not produce true potato seeds. “Dianshu47” (D47, *S. tuberosum*) and “Dianshu1418” (D1418, *S. tuberosum*) were selected for homologous grafting rootstock. These varieties produce a large number of flowers and true potato seeds. For heterologous grafting rootstock, “Zhongyan988” (ZY988, *S. lycopersicum*) tomato variety was purchased from Zhongyanyinong Seedling Technology Co., Ltd (http://www.bjzyyn.com/). The ungrafted and self-grafted Q9 plants were used as control. The plant materials were grown at the Teaching and Experimental Base of Yunnan Agricultural University, Kunming, Yunnan, China (latitude 25°08'N, longitude 102°45'E, altitude 1966 m).

### 2.2 Different grafting and cultivation approaches

Different grafting and cultivation methods were designed to control tuber yield (**Supplementary Figure S1**), including ungrafted (Q9), self-grafting (Q9/Q9), heterologous grafting (Q9/ZY988), intermediate rootstock grafting (Q9/ZY988/Q9), and heterologous grafting scion soil cover cultivation (Q9/ZY988-CS).

The same method was used for self-root grafting Q9/Q9 and homologous grafting Q9/D47 and Q9/D1418. The sprouted potato tubers were seeded in nursery seedling pots, and after about 2 weeks, non-hollowed Q9 potato shoots with 4–5 leaves and a height of 10 cm were selected as scions, non-hollowed Q9 and D47 and D1418 potato plants with a thick stem were selected as rootstock. The “cleft grafting” method was used for grafting (Wang et al., 2017; Zhang et al., 2019). Briefly, potato rootstock plants were cut approximately 3–5 cm above soil level, and a longitudinal cut of 1–2 cm was made at the center of the cut end of the potato rootstock shoot. Excess leaves of Q9 scions were removed to prevent transpiration, leaving only the top two leaves. The scions were given a wedge shape by symmetrically peeling the two sides of the base stem. Then, the scion was inserted into the cleft of the rootstock. Finally, the joints of the grafted plants were wrapped in plastic film.

For heterologous grafting, the tomato seeds were germinated on filter paper and then seeded in nursery seedling pots after 2 weeks. Healthy 4- to 5-week-old tomato seedlings with a stem thickness of 0.5–1 cm were used as rootstock. Fifteen days after the emergence of tomato seedlings, sprouted potatoes were seeded on a seedling tray and covered with 5 cm of soil. At approximately 2 weeks of age, non-hollowed potato shoots with 4–5 leaves and a height of 10 cm were selected as scions. The “cleft grafting” method was used for heterologous grafting, as described previously.

For intermediate rootstock grafting, we first grafted the tomato scion onto Q9 using the cleft grafting method (as described previously). After 10 days, another shoot or branch from Q9 was grafted onto the intermediate rootstock tomato (**Supplementary Figure S1D**).

The grafted plants were then grown for 1 week in an artificial climate chamber under low-light conditions (5000 Lux) with a photoperiod of 12 h and temperatures of 22°C (day) and 20°C (night) with a constant relative humidity of 85%–90%. Next, the viable grafted plants were planted in pots filled with soil (50% clay and 50% nursery substrate). In addition, we added 100 g of organic fertilizer and 20 g of controlled-release fertilizer, comprising nitrogen, phosphorus, and potassium in a 15:7:18 ratio, to each pot.

The cultivation method in the Q9/ZY988-CS group involved covering the stems of the scion with 10 cm of soil after heterologous grafting to promote tuberization in the scion (**Supplementary Figure S1E**).

### 2.3 Phenotypic analysis

We selected 15 plants from each group during the flowering period and recorded the number of flower buds, flowers, and fruit sets in the first inflorescence of the main stem. Next, we measured the fresh weights of different tissues of the grafted plants. In addition, pollen vitality was assessed using Alexander staining (Peterson et al., 2010).

### 2.4 Hybridization experiment

We first used Q9 as the male parent and D1418 as the female parent for crossing, at the same time, we used D1418 as the male parent with Q9 as the female parent for crossing. Then, the pollen from Q9/ZY988 group was used to cross with the female parent D1418. Briefly, the flower buds to be opened were emasculated the morning before pollination. The next day, we removed the anthers of the paternal plant, scraped off the pollen grains along the side of the pollen sac fissure with tweezers, and then evenly spread the pollen of the paternal plant on the stigma of the female parents. The stigma was covered with wheat straw to prevent the pollen from being blown off by the wind.

### 2.5 RNA extraction, sequencing, and RNA sequencing data processing

Total RNA from Q9/ZY988 scion pollen and controlled self-grafting Q9 pollen was extracted using a plant RNA extraction kit (Ambion, Thermo Fisher, Carlsbad, CA, USA). Sequencing libraries were generated using the NEBNext^®^ Ultra™ RNA Library Prep Kit for Illumina^®^ (NEB, Massachusetts, USA), following the manufacturer’s recommendations. The libraries were sequenced on an Illumina HiSeq platform.

The raw reads containing adapters, poly-N, and low-quality reads were filtered. The remaining clean reads of Q9/ZY988 and Q9 were aligned with the potato genome DM1-3_v6.1 (http://spuddb.uga.edu/index.shtml) using TBtools Hisat2-Align simple wrapper Plugin (Chen et al., 2020). After sequence alignments, the fragments per kilobase of transcript sequence per million (FPKM) method was used to calculate the gene expression levels (Trapnell et al., 2010). The differentially expressed genes (DEGs) between Q9/ZY988 and Q9 were analyzed using the DESeq R package (Wang et al., 2010). Genes with an adjusted P value of <0.05 and absolute log_2_ fold change value of ≥1.5, as determined by DESeq, were defined as DEGs. Gene ontology (GO) functional and KEGG pathway enrichment analyses were performed on DEGs using TBtools (Chen et al., 2020). The raw data generated in this study were uploaded to the Sequence Read Archive under BioProject No. PRJNA1039551 (https://www.ncbi.nlm.nih.gov/sra/PRJNA1039551).

### 2.6 Quantitative real-time PCR analysis

Real-time quantitative PCR (qRT-PCR) was performed using an Applied Biosystems 7500 real-time PCR instrument (Thermo Fisher, Carlsbad, CA, USA) and a two-step qPCR kit (PrimeScript™RT Reagent Kit and SYBR Premix ExTaq™, TaKaRa, Tokyo Metropolis, Japan). The PCR protocol was set as follows: 95°C for 7 min, followed by 39 cycles of 95°C for 15 s, 60°C for 30 s, and 72°C for 30 s. Total RNA was prepared from the freshly sampled potato pollens using the RNA simple Total RNA Kit (TIANGEN, Beijing, China). Then, cDNA was obtained via the reverse transcription of RNA samples using the TransScript^®^-Uni One-Step gDNA Removal and cDNA Synthesis SuperMix kit (TransGen Biotech Co., Ltd, Beijing, China), following the manufacturer’s protocol. The *StEF1α* gene was assessed as the internal reference gene. The primers used for the PCR are listed in **Supplementary Table S1**. Relative gene expression levels were calculated using the 2^−ΔΔCt^ method (Schmittgen and Livak, 2008).

### 2.7 Statistical analysis

At least three independent biological replicates were used for each analysis. All data were statistically analyzed using the SPSS 21.0 software (SPSS Inc., Chicago, IL, USA), and mean differences among the treatments were calculated using Duncan’s multiple range test. The bar and pie charts were plotted using the GraphPad 8.3.0 software. Differences with p <0.05 were considered statistically significant.

## 3 Results

### 3.1 Effects of grafting on potato fertility

**Figure 1:**
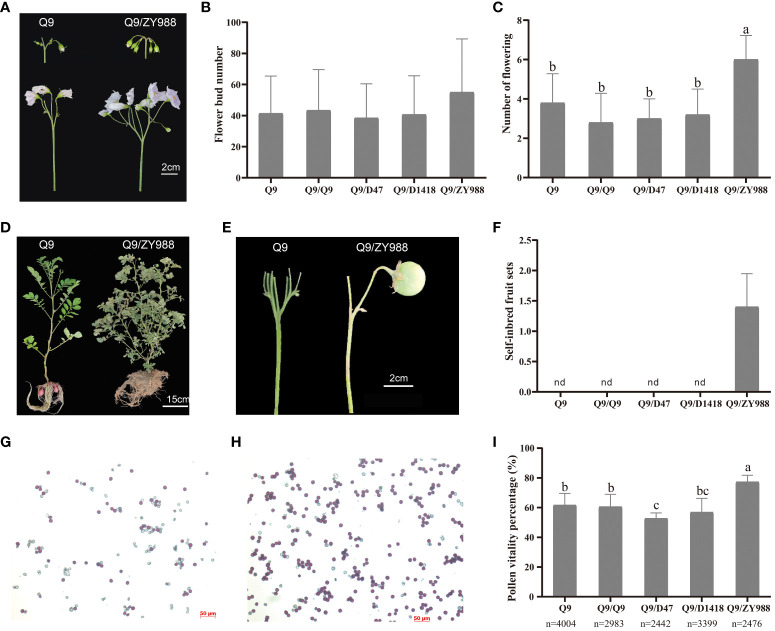
Effects of grafting on potato fertility. **(A)** Flower buds and open flowers of Q9 and Q9/ZY988. **(B)** The number of flower buds on the Q9 scion under different grafting treatments. **(C)** The number of flowers on the Q9 scion under different grafting treatments. **(D, E)** Fruit set phenotype of Q9 and Q9/ZY988 plants. **(F)** The number of self-inbred fruit sets on the Q9 scion under different grafting treatments. **(G, H)** Q9 and Q9/ZY988 pollen was subjected to Alexander staining and observed under a microscope. Red-colored pollens indicate strong vigor and light red and colorless pollens indicate no vigor or infertility (100×). **(I)** Pollen viability percentage for the Q9 scion under different grafting treatments. Q9, no grafting; Q9/Q9, self-grafting; Q9/D47, homologous grafting with Dianshu47; Q9/D1418, homologous grafting with Dianshu1418; Q9/ZY988, heterologous grafting with tomato; nd, not detected; the lowercase letters represent significant differences (t-test, p < 0.05); n, number of pollen grains.

To study the effects of different rootstocks on the fertility of the potato scion, we grafted Q9 scions on the rootstocks of high-fertility potato and tomato cultivars, namely, D47, D1418, and ZY988. All grafted plants exhibited normal growth and underwent a floral transition. In the flowering stage, the Q9/D1418 grafted plants exhibited the highest aboveground biomass, while the Q9/D47 and Q9/ZY988 plants exhibited lower aboveground biomass than the control plants (**Supplementary Table S2**). In the flower bud stage, compared to the other groups, the Q9/ZY988 plants exhibited less flower bud shedding, and most flowers in the inflorescence of these plants could open (**Figure 1A**). However, no significant differences in the total number of flower buds were observed among the groups (**Figure 1B**). In the peak flowering stage, compared to the ungrafted and self-grafted plants, the plants subjected to heterologous grafting exhibited significantly more number of flowers in the Q9 scion potato (**Figure 1C**). After the flowering period, it has been indicated by previous cultivation that no true potato seed could be formed in the case of normal planting Q9. The study observed the natural fecundity of Q9/ZY999 and found that all the heterografted potatoes produced true potato berries, and the flowers of the other grafting groups were aborted (**Figures 1D–F**), producing true potato seeds. Moreover, we observed a 15%–20% higher pollen viability in Q9/ZY988 plants than in ungrafted and self-grafted plants (**Figures 1G–I**). These findings showed that heterologous potato–tomato grafting effectively promoted flower development, improved pollen viability, and promoted the production of true potato seeds.

### 3.2 Effects of grafting the paternal fertility of Q9

Next, we assessed whether the enhanced pollen viability of the plants subjected to heterologous grafting improves scion fertility. Here, we used Q9 and D1418 as parents for cross-pollination. The plants produced from the cross between Q9 as the male parent and D1418 as the female parent did not generate any fruits. However, the cross between Q9 as the female parent and D1418 as the male parent successfully produced fruits. This finding indicated that the Q9 pollens might have impaired fertility. Furthermore, the cross between Q9/ZY988 as the male parent and D1418 as the female parent was able to generate fruits (**Table 1**). This finding showed that heterologous grafting enhanced the pollen fertility in the scion potato.

**Table 1:** Hybridization experiments.

Parentage	Crossing number	Fruit number	Fruit-setting rate (%)
D1418 × Q9	86	0	0
Q9 × D1418	87	3	3.4
D1418 × Q9/ZY988	195	8	4.1

### 3.3 Effects of tuber yield on pollen viability

**Figure 2:**
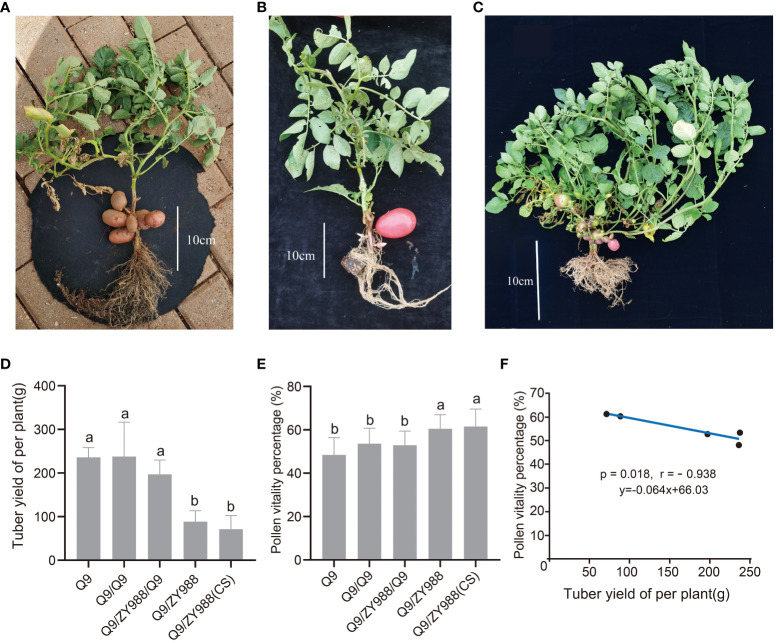
Tuber yield per plant and pollen viability percentage under different grafting and transplanting methods. **(A)** Tuberization of the heterologous grafting scion covering soil cultivation. **(B)** Tuberization of the plants subjected to intermediate rootstock grafting. **(C)** Tuberization of the plants subjected to heterologous grafting. These plants were prone to the formation of aerial tubers on the branches of the potato scion. **(D)** Histogram showing the tuber yield per plant for different plant groups. **(E)** Histogram showing the pollen viability percentage for different plant groups. **(F)** Scatter plots showing linear regression and R^2^ between pollen viability percentage and tuber yield per plant. The lowercase letters represent significant differences (t-test, p < 0.05).

Potato tubers are strong assimilate sinks and absorb assimilation products of the leaves, leaving insufficient assimilates for flower and pollen development. After potato/tomato heterologous grafting, rootstock tomato could not tuberize. In this study, we used potato/tomato heterologous grafting, intermediate rootstock grafting, and soil cover heterologous grafting plant scion to control tuber yield and ungrafted and self-grafted plants as control. As expected, the heterologous grafting scion soil cover cultivation (Q9/ZY988-CS) group plants (**Figure 2A**) and the intermediate rootstock grafting (Q9/ZY988/Q9) group plants (**Figure 2B**) with tuberizing, the heterologous grafting (Q9/ZY988) group plants does not tuberizing properly. However, Q9/ZY988 grafting promoted the formation of aerial potatoes on the branches of scion potatoes (**Figure 2C**). The tuber yield measurements revealed that the plants subjected to heterologous grafting and heterologous grafting scion soil cover cultivation exhibited significantly lower tuber yield than the control group (p < 0.05). The plants subjected to intermediate rootstock grafting also exhibited lower yield than the control plants; however, the difference was not significant (**Figure 2D**).

The scions of all grafted plants underwent a floral transition. Pollen viability assays showed significantly enhanced pollen viability in the plants subjected to heterologous grafting and heterologous grafting scion soil cover cultivation methods (**Figure 2E**). Interestingly, we found that the higher the tuber yield, the lower the pollen viability. Pearson’s correlation analysis revealed a significant negative correlation between pollen viability and tuber yield (p = 0.018, r = −0.938) (**Figure 2F**). These results indicated that there may be a presence of severe product assimilation competition between potato tubers and flower organs.

### 3.4 Pollen transcriptome

RNA-seq results are shown in **Supplementary Table S3**. There were more than 60 million raw data points per sample. Quality control revealed 9.22 to 10.09 Gb of clean bases with a Q30 ratio of ~91%. A GC content of over 41.11% was obtained from each of the six libraries. The Q9 and Q9/ZY988 transcriptome libraries were mapped to the DM_v6.1 *S. tuberosum* reference genome, achieving a total mapping rate of 87.68%–88.63% (**Supplementary Table S2**). These results showed that our sequencing data had a high depth of coverage in the genome and that the data were accurate and sufficient for subsequent bioinformatic analyses.

**Figure 3:**
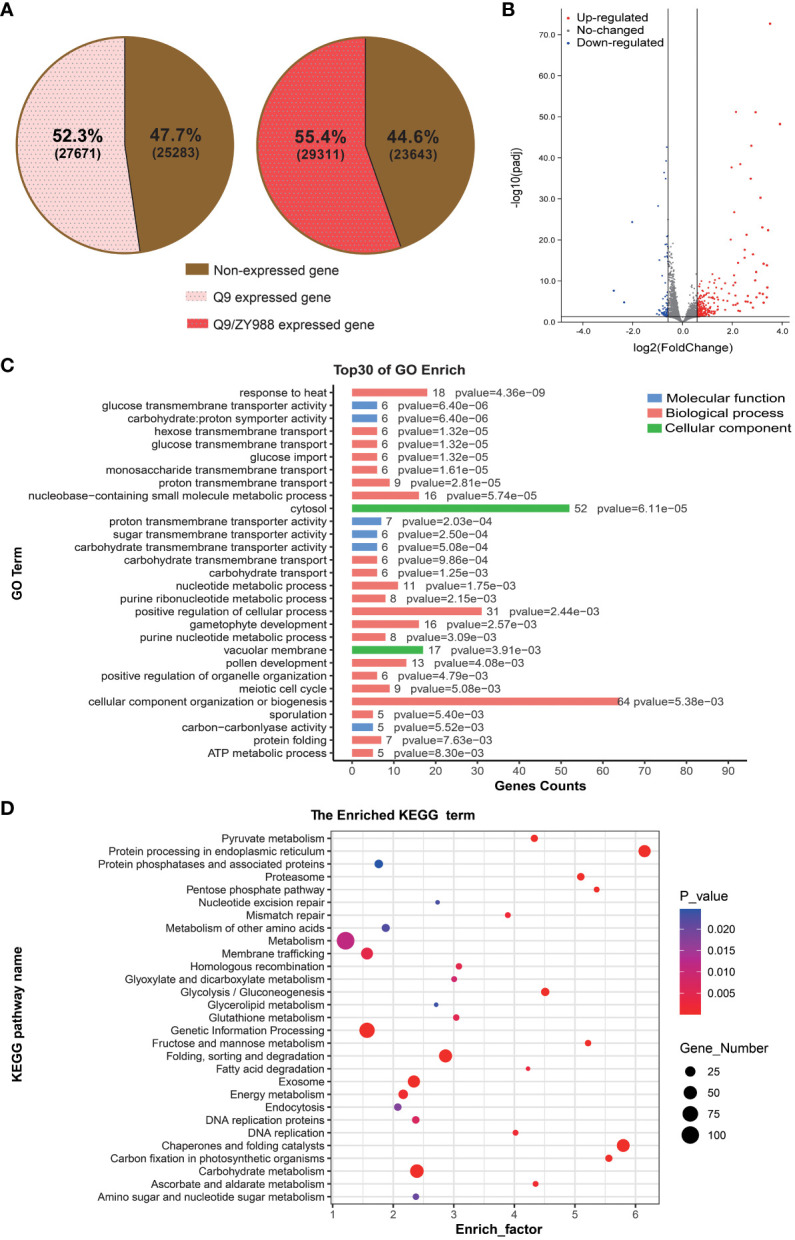
Analysis of differentially expressed genes (DEGs), GO terms, and KEGG pathway enrichment between Q9/ZY988 and Q9. **(A)** Q9 and Q9/ZY988 samples had the proportion of genes with read counts to all annotated genes in the reference genome. **(B)** Volcano plots of DEGs between Q9/ZY988 and Q9, log_2_ fold change >0.5, p <0.05. **(C)** GO analysis of DEGs. **(D)** KEGG pathway enrichment analysis of DEGs.

We detected 27,671 and 29,311 genes with read counts from Q9 and Q9/ZY988 pollens, covering 53.3% and 55.4% of all the annotated genes in the reference genome, respectively (**Figure 3A**). A total of 513 DEGs were obtained between Q9 and Q9/ZY988, including 380 upregulated and 133 downregulated genes in Q9/ZY988 (**Figure 3B**).

We annotated these DEGs using GO analysis to evaluate their functions. The DEGs were primarily enriched in stress response processes, including responses to biological or abiotic stimuli, such as temperature, light, metal ions, fungi, and hormones. Some DEGs were also significantly enriched in carbohydrate synthesis and transport and gametophyte and pollen development (**Figure 3C**).

Furthermore, we performed a KEGG pathway enrichment analysis for pathway enrichment. The DEGs significantly enriched in metabolic processes such as protein folding, sorting, and degradation; protein processing in the endoplasmic reticulum; chaperone and folding catalysts; carbohydrate metabolism; glucose metabolism; and amino acid metabolism, consistent with the results of the GO enrichment analysis (**Figure 3D**). These results indicated that heterologous grafting significantly impacted the protein processing and carbohydrate metabolism pathways.

### 3.5 Functional analysis of DEGs

**Figure 4:**
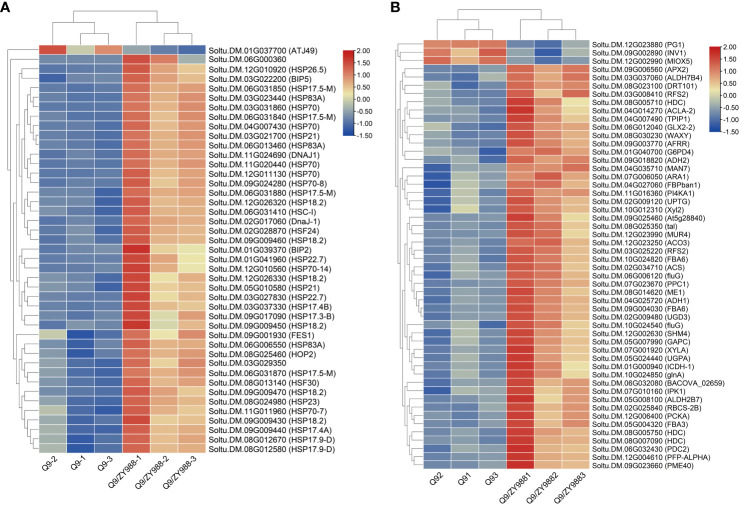
Heat map of the expression levels of the DEGs associated with the heat-shock protein family and carbohydrate metabolism. **(A)** Genes belonging to the heat-shock protein family. **(B)** Genes related to carbohydrate metabolism.

GO and KEGG analyses primarily enriched the DEGs into stress response and protein-related pathways, respectively. This is mainly due to a significant upregulation of the genes associated with stress response in pollen (**Figure 4A**). Notably, apart from the stress response, these gene products also participate as molecular chaperones in the deep processing of several other proteins, including protein folding, sorting, and degradation in the endoplasmic reticulum and Golgi apparatus. Thus, these DEGs promoted rapid protein synthesis and high physiological activity in fertile pollens (Miernyk, 1999). Furthermore, we also observed an upregulation of the genes enriched in carbohydrate metabolism. Hence, heterologous grafting also promoted carbohydrate metabolism within pollen cells (**Figure 4B**).

Furthermore, GO enrichment revealed 18 DEGs to be associated with gametophyte and pollen development (**Table 2**). These genes have previously been shown to be associated with embryonic development, blastocyst development, pollen development, mitosis during pollen development, pollen cell wall development, and gamete fusion. These results showed that heterologous grafting improved pollen fertility by promoting the expression of genes related to pollen development.

**Table 2:** DEGs related to gametophyte and pollen development.

Gene ID	Log2FC	Padj	Description of the encoded protein
Soltu.DM.03G010420	0.88696	2.85E−07	Ribosomal RNA processing 5, RRP5
Soltu.DM.03G032940	0.719575	3.07E−07	RAB geranyl transferase beta subunit 1, RGTB1
Soltu.DM.01G041360	0.838858	0.000096	Transducin/WD40-2 protein, tstF
Soltu.DM.03G025120	1.100372	0.000277	Cysteine endopeptidase 1, CYSEP1
Soltu.DM.03G034900	0.712357	0.000466	Regulator of telomere elongation helicase 1, RTEL1
Soltu.DM.06G014180	0.581838	0.000786	Flowering time control protein, FPA
Soltu.DM.05G024440	1.070433	0.003034	UDP-glucose pyrophosphorylase 2, UGPA
Soltu.DM.12G002630	1.061142	0.004006	Serine hydroxymethyltransferase 4-like, SHM4
Soltu.DM.02G009120	0.91567	0.004788	Alpha-1,4-glucan-protein synthase, UPTG
Soltu.DM.01G021510	0.555083	0.006723	Gamete expressed 2, GEX2
Soltu.DM.03G034780	0.624576	0.007148	Regulator of telomere elongation helicase 1 homolog isoform X2, RTEL1
Soltu.DM.01G051700	0.679792	0.008369	Erwinia-induced protein 1 precursor, LYM2
Soltu.DM.03G032800	0.96871	0.009185	RAB geranyl transferase beta subunit 1, RGTB1
Soltu.DM.01G043650	−0.5424	0.01365	Multicopy suppressor of IRA 1, MSI1
Soltu.DM.08G022380	1.039917	0.015502	Plant-specific negative regulator of the APC/C complex, SAMBA
Soltu.DM.07G023520	0.68417	0.018443	Tubby-like F-box protein 7, TULP7
Soltu.DM.05G006220	0.560042	0.020187	Myb-related protein A isoform X2, MYB88
Soltu.DM.01G029410	1.3013728	5.77E−11	Late embryogenesis abundant protein 6-like, LEA6

### 3.6 Validation of RNA-seq data using qRT-PCR

**Figure 5:**
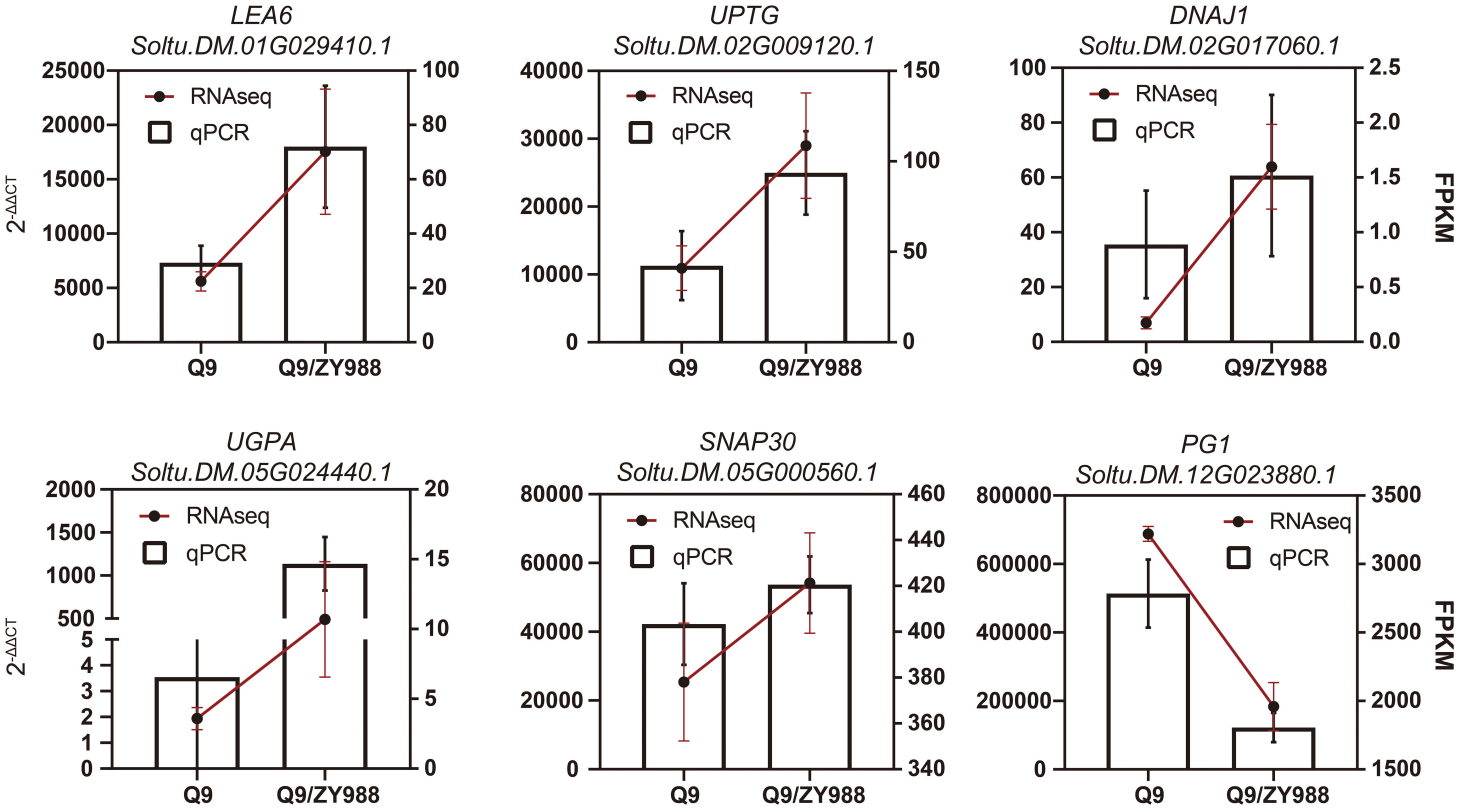
Expression analysis of six DEGs between Q9 and Q9/ZY988. The bar graph represents the RT-qPCR data, and the results are derived using the 2^−ΔΔCt^ method. The line chart represents the RNA-seq data, and the results are expressed in fragments per kilobase of transcript sequence per million (FPKM).

To validate the quality of RNA-seq data, we selected and analyzed six genes related to carbohydrate metabolism, protein folding, and gametophyte development in Q9 and Q9/ZY988 using qRT-PCR. These genes included Soltu.DM.02G009120.1, Soltu.DM.05G024440.1, Soltu.DM.12G023880.1, Soltu.DM.02G017060.1, Soltu.DM.01G029410.1, and Soltu.DM.05G000560.1 encoding alpha-1,4-glucan-protein synthase (UPTG), UDP-glucose pyrophosphorylase (UGPA), polygalacturonase (PG1), DNAJ heat shock protein 1 (DNAJ1), late embryogenesis abundant protein 6 (LEA6), and soluble N-ethylmaleimide-sensitive factor adaptor protein 30 (SNAP30), respectively. We observed a significant upregulation of *LEA6*, *UPTG*, *DNAJ1*, *UGPA*, and *SNAP30* and a significant downregulation of *PG1* in Q9/ZY988. These findings were consistent with the results of the transcriptomic analysis, validating the reliability of the transcriptome data (**Figure 5**) and the expression profiles derived from the RNA-seq data.

## 4 Discussion

In this study, we performed homologous grafting between the poor-fertility potato variety Q9 and high-fertility potato varieties D47 and D1418. However, it did not impact the flowering, fruit formation, and pollen vitality of the Q9 scion. In addition, we performed heterologous grafting between Q9 and a tomato variety ZY988 and observed markedly improved flower and fruit development in the Q9 scion. These findings were consistent with the results of the previous studies on potato/tomato (Thijn, 1954; Jiang, 2016; Takeuchi et al., 2022), potato/*Physali alkekengi* (Qi, 2015), and potato/*Datura stramonium* heterologous grafting (Zhang et al., 2022). Pollen viability is a prerequisite for sexual reproduction in plants. In the hybrid experiment, it was proved that the sterility of Q9 was due to pollen abortion, and potato/tomato heterologous grafting could solve this problem. This method is useful for potato breeding.

The simultaneous development of flowers and tubers (Jansky and Thompson, 1990) and inadequate energy supply impair the development of male gametes, leading to male infertility (Chen and Liu, 2014). Previous studies have shown that both flowering and fruiting significantly reduce vegetative growth and tuber yield in some potato cultivars (Bartholdi, 1942). The method of stolon pruning, girdling cutting to remove the stem cortex, and use steel wire to tie the base of the stem can restricts carbohydrate transport to the tuber, and subsequently, promotes flowering and improves pollen quality (McLean and Stevensen, 1952; Thijn, 1954; Jessup, 1958). In the present study, we observed a significant negative correlation between tuber yield and pollen. However, previous studies reported no correlation between flower production and tuber yield, and fruit setting was more dependent on the internal and external environments of the plant (Werner, 1934). In most potato cultivars, tuber yields were not affected by fruiting, except for a few varieties with high tuber yields (HM et al., 1975). These findings indicated that the influence of fruiting on tuber yield might vary across different genotypes. Thus, the distribution of assimilation products between potato tubers and flowers warrants further research.

RNA-seq revealed 513 DEGs between Q9 and Q9/ZY988 pollen samples. The functional analysis of these DEGs showed that they were primarily associated with deep protein processing, such as protein folding, sorting, and degradation. Among these DEGs, we detected an upregulation of genes encoding heat shock proteins (HSPs). This finding was consistent with the results of a previous study reporting an upregulation of the HSP gene family in Arabidopsis during the development of pollen and pollen tube (Wang et al., 2008). HSPs have been shown to function as chaperones regulating protein modification during pollen development, which is associated with high physiological activity in fertile pollen (Miernyk, 1999; Fragkostefanakis et al., 2016). In addition, in the current study, the DEGs were enriched in several carbohydrate metabolic processes, including glycolysis, fructose metabolism, mannose metabolism, and pyruvate metabolism. For example, the *phosphoenolpyruvate carboxylase 1* (*PPC1*) gene, which encodes phosphoenolpyruvate carboxylase (PEPC), catalyzes the synthesis of oxaloacetic acid and promotes the accumulation of stored substances in pollen during maturation (Igawa et al., 2010). Protein targeting to glycogen (PTG) participates in the biosynthesis of polysaccharides, development of microspores, and pollen mitosis (Drakakaki et al., 2006). Knockout of UDP-glucose pyrophosphorylase (*UDPA*), which encodes glucose pyrophosphate carboxylase in *Arabidopsis*, has been shown to decrease cellulose and callosum synthesis, resulting in male infertility (Park et al., 2010). Another study on anther metabolomes for male sterile and fertile potatoes reported significant differences in their carbohydrate accumulation and high levels of amino acids in the anthers of the sterile genotype, which might be attributed to impaired carbohydrate and fatty acid metabolism (Shishova et al., 2019).

Furthermore, the plants subjected to heterologous grafting exhibited significant alterations in the expressions of genes associated with gametophyte and pollen development, such as *RGTB1* (Gutkowska et al., 2015), *SAMBA* (Eloy et al., 2012) and *GEX2* (Mori et al., 2014). These genes play essential roles in the regulation of pollen development and the double fertilization process and might regulate pollen viability during potato/tomato heterologous grafting.

## 5 Conclusion

In this study, homologous grafting between potato varieties did not effectively improve scion fertility; however, heterologous potato/tomato grafting promoted flowering and fruit formation in potato scion. In addition, potato/tomato heterologous grafting significantly improved the pollen viability of scion potatoes and was associated with the absence of potato tubers. Heterologous grafting also promoted the transcription of genes related to protein processing, carbohydrate metabolism, and pollen development, resulting in the production of fertile pollen.

## Data availability statement

The datasets presented in this study can be found in online repositories. The names of the repository/repositories and accession number(s) can be found in the article/**Supplementary Material**.

## Author contributions

XZ: Investigation, Methodology, Writing – original draft, Data curation, Visualization. LB: Data curation, Writing – original draft, Formal analysis, Methodology. ML: Software, Visualization, Writing – review & editing. YL: Data curation, Writing – review & editing. RH: Resources, Writing – review & editing. HG: Funding acquisition, Writing – review & editing, Project administration, Resources.
