# Supplementary material for: Pollen transcriptomic analysis provided insights into understanding the molecular mechanisms underlying grafting-induced improvement in potato fertility
Source: Front Plant Sci. 2024 Mar 28;15:1338106. doi: 10.3389/fpls.2024.1338106 (PMC11007164; doi:10.3389/fpls.2024.1338106)
Supplement: Supplementary file 1 [file DataSheet_1.docx]

Supplementary Material

# Supplementary Figures


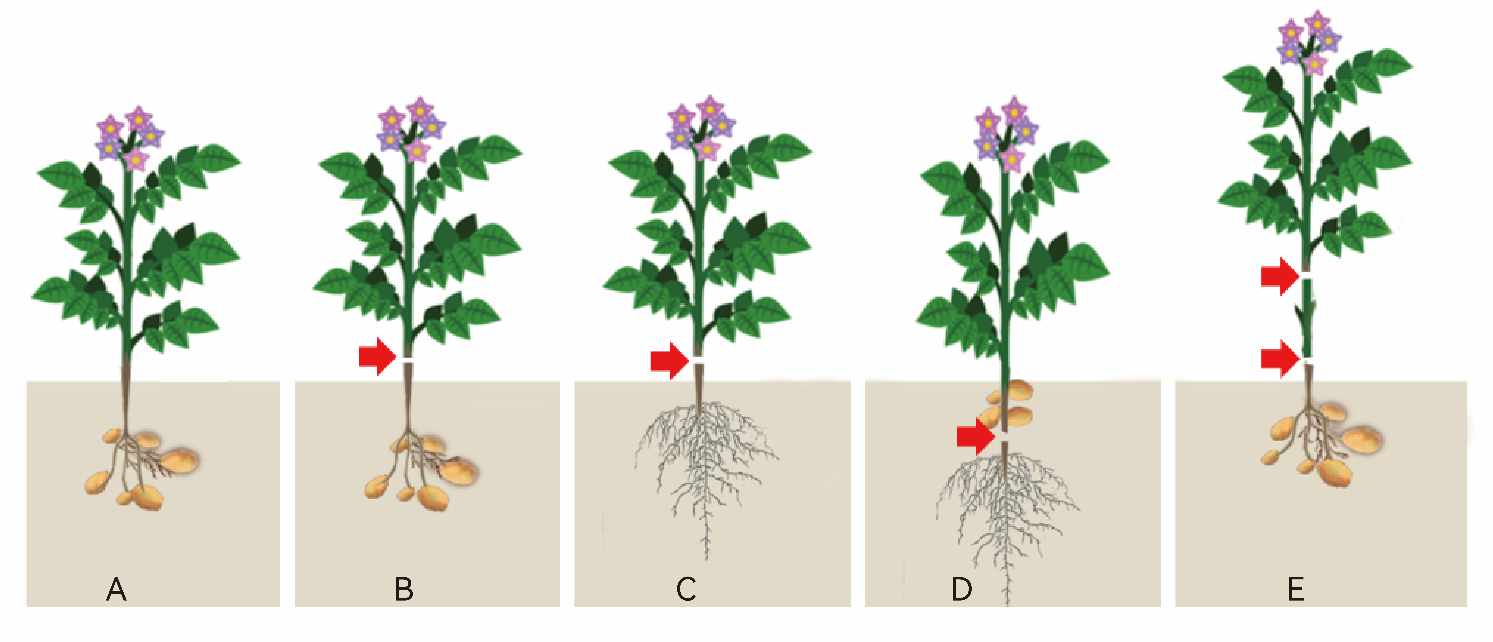


**Supplementary Figure 1.** **Schematic diagram of different grafting and transplantation methods employed in this study.**

**(A)** No grafting (Q9). **(B)** Self-grafting (Q9/Q9). **(C)** Heterologous grafting (Q9/ZY988). **(D)** Heterologous grafting scion covering soil cultivation (Q9/ZY988-CS). **(E)** Intermediate rootstock grafting (Q9/ZY988/Q9). The red arrows point to the grafting sites.

# Supplementary Tables

**Supplementary Table 1.** Primers used for real-time quantitative PCR

| Primer name | Sequence (5'-3') |
| --- | --- |
| StEF1α_F | ATTGGAAACGGATATGCTCCA |
| StEF1α_R | TCCTTACCTGAACGCCTGTCA |
| LEA6_qF | TTTACATGTCAACAAAGCAGCACAG |
| LEA6_qR | GAGGTGAGTGTTGGTGAAGC |
| UPTG_qF | GGTCCTAGATCCTCTTGCATTTCC |
| UPTG_qR | AACAAAGCAATCATCATCAATGGTG |
| DNAJ1_qF | GTTGATAAGAGTGCTACAGATGATG |
| DNAJ1_qR | CCCATACTGATCATAAATTGCCC |
| UGPA_qF | CGACAAGTTAGCACCTCTCTCTG |
| UGPA_qR | GGAATGTCAAACCATTACGAACTTC |
| SNAP30_qF | ATCAGCCTCTAGTACACCTCCTC |
| SNAP30_qR | TCACCCAATATGTTGCTCAAATCC |
| PG1_qF | GCTGATATTAGTGAGGCTGTGGG |
| PG1_qR | GCATGGTCCTTCTAATTTCACTTGG |

**Supplementary Table 2.** Effects of different grafting methods on plant biomass

Note: Different letters indicate significant differences in different graft groups

| Treatment | Root weight  (g) | | Stem weight  (g) | Leaf weight  (g) | | Tuber weight  (g) | |  |
| --- | --- | --- | --- | --- | --- | --- | --- | --- |
| Q9  Q9**/**Q9  Q9**/**D47  Q9**/**D1418  Q9/ZY988 | 18.63±3.74 b  32.43±0.63 a  22.70±8.21 ab  25.7±4.74 ab  30.20±4.35 ab | 427.6±63.11 b  410.73±52.06 b  277.7±72.68 b  707±146.68 a  298.6±28.68 b | | | 140.83±61.10 b  145.8±16.17 b  93.1±18.55 b  270.4±21.05 a  103±19.15 b | | 493.50±62.11 a  489.10±88.16 a  293.63±114.55 a  382.80±90.75 a  - | |

**Supplementary Table 3.** Sample libraries derived from sequencing data

| Sample name | Raw reads | Clean reads | Clean bases | Q30 (%) | GC (%) | Multiple mapped (%) | Unique mapped (%) | Total mapped (%) |
| --- | --- | --- | --- | --- | --- | --- | --- | --- |
| Q9-1 | 65395524 | 65382756 | 9.74G | 91.9 | 41.11 | 2.62 | 85.53 | 88.16 |
| Q9-2 | 61870706 | 61859074 | 9.22G | 92.06 | 41.13 | 3.84 | 83.84 | 87.68 |
| Q9-3 | 65784034 | 65771550 | 9.8G | 91.49 | 41.25 | 3.46 | 84.64 | 88.10 |
| Q9/ZY988-1 | 67202524 | 67195082 | 10.03G | 91.39 | 41.42 | 3.75 | 84.88 | 88.63 |
| Q9/ZY988-2 | 67685384 | 67672888 | 10.09G | 91.57 | 41.12 | 2.99 | 84.73 | 87.72 |
| Q9/ZY988-3 | 65660004 | 65650228 | 9.8G | 90.94 | 41.44 | 2.84 | 85.61 | 88.45 |
